# Supplementary material for: Recommendations of high-quality clinical practice guidelines related to the process of starting dialysis: A systematic review
Source: PLoS One. 2022 Jun 13;17(6):e0266202. doi: 10.1371/journal.pone.0266202 (PMC9191707; doi:10.1371/journal.pone.0266202)
Supplement: S1 Appendix — (PDF) [file pone.0266202.s001.pdf]

S1 Appendix: Search strategy

Search strategy Medline: ((((((((("kidney failure, chronic"[MeSH Terms] OR chronic kidney failure[Title/Abstract]) OR "kidney failure, chronic"[MeSH Terms]) OR chronic renal failure[Title/Abstract]) OR end stage kidney disease[Title/Abstract]) OR end-stage kidney disease[Title/Abstract]) OR chronic kidney disease[Title/Abstract]) OR ("renal dialysis"[MeSH Terms] OR "dialysis"[MeSH Terms])) OR dialysis[Title/Abstract]) OR renal replacement therapy[Title/Abstract]) OR "renal replacement therapy"[MeSH Terms]) AND (Guideline[ptyp] OR Practice Guideline[ptyp])

Search strategy Lilacs: (tw:(dialisis)) OR (tw:(enfermedad renal cronica)) OR (tw:(tratamiento renal sustitutivo)) OR (tw:(insuficiencia renal cronica)) FILTERED BY GUIDELINE

Search strategy EMBASE: (kidney or renal or dialysis).ti. and guideline*.ti.

Search strategy Web of Science: (renal OR kidney OR dialysis)[TITLE] AND (guideline OR position)[TITLE]
