# Supplementary material for: Recommendations of high-quality clinical practice guidelines related to the process of starting dialysis: A systematic review
Source: PLoS One. 2022 Jun 13;17(6):e0266202. doi: 10.1371/journal.pone.0266202 (PMC9191707; doi:10.1371/journal.pone.0266202)
Supplement: S1 Table — (PDF) [file pone.0266202.s002.pdf]

S2 Table: Excluded CPG with scores in domains 3 or 6 less than 60% in the AGREE-II instrument

Organization (Year of publication)	Name	Domain scores (%)						Intraclass correlation coefficient (95% CI)
		Scope and Purpose	Stakeholder involvement	Rigour of development	Clarity and presentation	Applicability	Editorial independence	
Canadian Society of Nephrology (2008)	Guidelines for the management of chronic kidney disease	94	64	55	100	19	88	0.87 (0.42-0.98)
Japanese Society for Dialysis Therapy (2009)	Guidelines for Peritoneal Dialysis	94	56	26	100	15	8	0.64 (0.12-0.94)
Renal Physician Association. Agency for Healthcare and Quality Research, (2010)	Guideline recommendations and their rationales for the treatment of adult patients. In: Shared decision-making in the appropriate initiation of and withdrawal from dialysis, 2nd edition.	94	100	92	94	56	50	0.97 (0.86-0.99)
ERBP Advisory Board (2011)	When to start dialysis: updated guidance following publication of the Initiating Dialysis Early and Late (IDEAL) study	67	28	31	92	19	71	0.70 (0.19-0.95)
AIDS Working Group of SEIMC, SEN; SEQC (2014)	Consensus document on the management of renal disease in HIV-infected patients	89	75	38	86	15	54	0.74 (0.53-0.96)
Ministry of Public Health, Qatar (2016)	Chronic kidney disease in adult	81	67	49	92	15	63	0.60 (0.20-0.93)
Ministry of Health and Labour and Welfare, and the Japanese Society of Nephrology (2016)	Evidence-based clinical practice guidelines for polycystic kidney disease	100	44	33	97	8	79	0.53 (0.29-0.92)
Society of Nephrology, Hong-Kong (2019)	Clinical practice guidelines for the provision of renal service in Hong Kong	92	72	26	100	27	42	0.93 (0.63-0.99)
ADPKD Clinical Guideline Working Group (2020)	Clinical Practice Guideline for Autosomal Dominant Polycystic Kidney Disease in China	42	0	16	94	0	75	0.60 (0.19-0.93)
Asian Pacific Society of Nephrology (2021)	Clinical Practice Guideline on Diabetic Kidney Disease	97	36	43	100	50	21	0.91 (0.58-0.99)

ERBP: European Renal Best Practice; SEIMC: Spanish Society of Infectious Diseases and Clinical Microbiology; SEN: Spanish Society of Nephrology; SEQC: Spanish Society of Clinical Chemistry and Molecular Pathology; ADPKD: Autosomal dominant polycystic kidney disease
