# Supplementary material for: Recommendations of high-quality clinical practice guidelines related to the process of starting dialysis: A systematic review
Source: PLoS One. 2022 Jun 13;17(6):e0266202. doi: 10.1371/journal.pone.0266202 (PMC9191707; doi:10.1371/journal.pone.0266202)
Supplement: S2 Table — (PDF) [file pone.0266202.s003.pdf]

S3 Table: Complete scores of high-quality CPGs according to the AGREE II instrument

Organization	Name	Domain scores (%) Scope and Purpose	Stakeholder involvement	Rigour of development	Clarity and presentation	Applicability	Editorial independence	Intraclass correlation coefficient (95% CI)	Overall	Recommended
Chile Ministry of Health	Clinical guideline: peritoneal dialysis	97	58	68	100	71	96	0.94 (0.71-0.99)	6	Yes
KDIGO	KDIGO 2012 CPG for the Evaluation and Management of CKD	100	92	90	100	46	92	0.98 (0.92-0.99)	7	Yes
UK Renal Association	Planning, Initiating and Withdrawal of Renal Replacement Therapy	72	42	60	97	44	63	0.88 (0.46-0.98)	5	Yes
Canadian Society of Nephrology	CPG for timing the initiation of chronic dialysis	100	75	89	100	96	92	0.97 (0.85-0.99)	7	Yes
ERBP	CPG on the management of patients with diabetes and CKD stage 3b or higher	100	100	100	100	58	100	0.95 (0.73-0.99)	7	Yes
National Kidney Foundation KDOQI	KDOQI CPG for Haemodialysis Adequacy: 2015 update	86	39	82	97	17	71	0.96 (0.81-0.99)	5	Yes
KHA - CARI	Autosomal Dominant Polycystic Kidney Disease Guideline: Management of ESKD	81	89	77	100	35	83	0.89 (0.50-0.98)	5	Yes
Spain Ministry of Health	CPG on detection and management of CKD	100	97	97	100	81	92	0.99 (0.97-0.99)	7	Yes
ERBP	CPG on the management of older patients with CKD stage 3b or higher	100	94	100	100	58	96	0.99 (0.98-0.99)	7	Yes
UK Renal Association	CPG Peritoneal Dialysis in Adults and Children	81	78	60	97	38	79	0.76 (0.11-0.96)	5	Yes
NICE	Renal replacement therapy and conservative management	97	94	96	100	96	96	0.99 (0.98-0.99)	7	Yes
International Society of Peritoneal Dialysis	Prescribing High Quality Goal-Directed Peritoneal Dialysis	100	97	64	100	42	92	0.80 (0.20 – 0.97)	6	Yes
	**Median scores (range)**	93 (72-100)	80 (39-100)	82 (60-100)	99 (97-100)	57 (17-96)	88 (63-100)			
