# Supplementary material for: Recommendations of high-quality clinical practice guidelines related to the process of starting dialysis: A systematic review
Source: PLoS One. 2022 Jun 13;17(6):e0266202. doi: 10.1371/journal.pone.0266202 (PMC9191707; doi:10.1371/journal.pone.0266202)
Supplement: S3 Table — (PDF) [file pone.0266202.s004.pdf]

S4 Table: Main objective of included high-quality CPGs

Name, organization, and year of publication	Main objective of CPG (extracted as stated on the document)
**Clinical guideline: peritoneal dialysis**<br>(Chile Ministry of Health, 2010)[15]	This guideline provides recommendations, based on the best level of evidence, aimed at optimising the management of patients who enter a PD program, so that they are treated under known and duly registered standards and protocols.
**CPG for the Evaluation and Management of CKD**<br>(KDIGO, 2013)[16]	The goal of this guideline is to clarify the definition and classification system of CKD, and to develop appropriate guidance as to the management and care of people with CKD (who are not on RRT). In addition, we present a framework which should foster an extended collaborative research
**Planning, Initiating and Withdrawal of RRT**<br>(UK Renal Association, 2013)[17]	This guideline provides recommendations on best practice in the planning, initiating and withdrawal of renal replacement therapy.
**CPG for timing the initiation of chronic dialysis**<br>(Canadian Society of Nephrology, 2014)[18]	Considering the enormous burden imposed by dialysis on patients and health care systems, there is a need for a judicious approach to dialysis initiation.
**CPG on management of patients with diabetes and CKD stage 3b or higher**<br>(ERBP, 2015)[19]	This clinical practice guideline was designed to facilitate informed decision-making on the management of adult individuals with diabetes mellitus and CKD stage 3b or higher (eGFR <45 mL/min). It was not intended to define a standard of care and should not be construed as such. It should not be interpreted as a prescription for an exclusive course of management.
**Clinical Practice Guideline for Haemodialysis Adequacy: 2015 update**<br>(National Kidney Foundation KDOQI, 2015)[20]	The 2015 update of the KDOQI Clinical Practice Guideline for HD Adequacy is intended to assist practitioners caring for patients in preparation for and during HD.
**ADPKD Guideline: Management of End-Stage Kidney Disease**<br>(KHA – CARI, 2015)[21]	This guideline outlines the evidence base for outcomes related to end-stage kidney disease (ESKD) specific to ADPKD.
**CPG on detection and management of CKD**<br>(Spain Ministry of Health, 2016)[22]	This clinical practice guideline aims to serve as an instrument to reduce uncertainty and variability in the detection and management of people with chronic kidney disease.
**CPG on management of older patients with CKD stage 3b or higher**<br>(ERBP, 2016)[23]	The guideline specifically covers management of older (>65 years of age) patients with CKD stage 3b or higher (eGFR <45 mL/min/1.73 m2), with a focus on six major areas: (1) estimation of GFR for classification and drug dose adaptation; (2) prognosticating rate of progression to end-stage renal disease; (3) prognosticating risk of death in the medium term; (4) assessment of functional status and strategies to improve it; (5) assessment of nutritional status and strategies to improve it; (vi) appraisal of benefits and drawbacks of RRT versus conservative care.
**CPG Peritoneal Dialysis in Adults and Children**<br>(UK Renal Association, 2017)[24]	These guidelines cover the organisation and performance of PD as a treatment for kidney patients, including infants and children.
**Renal replacement therapy and conservative management**<br>(NICE, 2018)[25]	This guideline covers renal replacement therapy (dialysis and transplantation) and conservative management for people with chronic kidney disease stages 4 and 5. It aims to improve quality of life by making recommendations on planning, starting and switching treatments, and coordinating care.
**Prescribing High Quality Goal-Directed Peritoneal Dialysis**<br>(International Society of Peritoneal Dialysis, 2020)[26]	This guideline has been written with the focus on the person doing PD. It is proposed that dialysis delivery should be 'goal-directed'. This involves discussions between the person doing PD and the care team (shared decision-making) to establish care goals for dialysis delivery. The aims of these care goals are (1) to allow the person doing PD to achieve his/her own life goals and (2) to promote the provision of high-quality dialysis care by the dialysis team.
