# Supplementary material for: Recommendations of high-quality clinical practice guidelines related to the process of starting dialysis: A systematic review
Source: PLoS One. 2022 Jun 13;17(6):e0266202. doi: 10.1371/journal.pone.0266202 (PMC9191707; doi:10.1371/journal.pone.0266202)
Supplement: S4 Table — (PDF) [file pone.0266202.s005.pdf]

S5 Table: Complete text of recommendations related to the start of dialysis

Clinical Practice Guideline	Theme 1: timing of initiation of dialysis
**Chile Ministry of Health (2010)**	-
**KDIGO (2013)**	We suggest that dialysis be initiated when one or more of the following are present: symptoms or signs attributable to kidney failure (serositis, acid-base or electrolyte abnormalities, pruritus); inability to control volume status or blood pressure; a progressive deterioration in nutritional status refractory to dietary intervention; or cognitive impairment. This often but not invariably occurs in the GFR range between 5 and 10 ml/min/1.73 m2. (2B)
**UK Renal Association (2013)**	We recommend that the decision to start RRT in patients with CKD stage 5 (eGFR < 15ml/min/1.73m2) should be based on a careful discussion with the patient of the risks and benefits of RRT taking into account the patients‘ symptoms and signs of renal failure, nutritional status, co-morbidity, functional status, and the physical, psychological and social consequences of starting dialysis in that individual (1D)
**Canadian Society of Nephrology (2014)**	For adults (aged > 18 yrs) with an eGFR of less than 15 mL/min per 1.73 m2, we recommend an intent-to-defer over an intent-to-start-early approach for the initiation of chronic dialysis. (Strong recommendation; moderate-quality evidence.)<br><br>Favour of a strong recommendation for an intent-to-defer approach to dialysis. An intent-to-start-early strategy is not justified given the lack of compelling benefit, along with the additional burden to patients and the health care system. An intent-to-defer strategy requires that patients be closely monitored for uremic symptoms or other complications, or a decline in eGFR to 6 mL/ min per 1.73 m2 or less, which would serve as indications for starting dialysis.
**ERBP (2015)**	We recommend initiating dialysis in patients with diabetes on the same criteria as in patients without diabetes (1A).
**National Kidney Foundation KDOQI (2015)**	The decision to initiate maintenance dialysis in patients who choose to do so should be based primarily upon an assessment of signs and/or symptoms associated with uremia, evidence of protein-energy wasting, and the ability to safely manage metabolic abnormalities and/or volume overload with medical therapy rather than on a specific level of kidney function in the absence of such signs and symptoms. (Not Graded)
**KHA – CARI (2015)**	-
**Spain Ministry of Health (2016)**	-
**ERBP (2016)**	-
**UK Renal Association (2017)**	-
**NICE (2018)**	Consider starting dialysis when indicated by the impact of symptoms of uremia on daily living, or biochemical measures or uncontrollable fluid overload, or at an estimated glomerular filtration rate (eGFR) of around 5 to 7 ml/min/1.73m2 if there are no symptoms.<br><br>Ensure the decision to start dialysis is made jointly by the person (or, where appropriate, their family members or carers) and their healthcare team.<br><br>Before starting dialysis in response to symptoms, be aware that some symptoms may be caused by non-renal conditions.<br><br>Involve the person and their family members or carers (as appropriate) in shared decision-making over the course of assessment to include: clinical preparation; psychosocial evaluation, preparation and support; the person's individual preferences for type of RRT and when to start; how decisions are likely to affect daily life.
**International Society of Peritoneal Dialysis (2021)**	-

Clinical Practice Guideline	Theme 2: selection of dialysis modality
**Chile Ministry of Health (2010)**	The informed and reasoned choice made by the patient and the family must be the key element of the decision. If done so, most patients would choose PD over HD (evidence B)<br><br>PD is indicated for every CKD-stage 5 patient, except for those who have a clear contraindication. (recommendation C)
**KDIGO (2013)**	-
**UK Renal Association (2013)**	We suggest that all patients should be encouraged to perform home dialysis therapy where possible, as part of an integrated approach to RRT (2B).<br><br>We suggest that where home dialysis is not possible, patients and their carers/partners should be actively involved in their dialysis treatment, be encouraged to perform as much self-care as possible and be engaged in all aspects of their treatment including medicines management and changes in diet and lifestyle (2B).<br><br>We suggest that pre-renal replacement therapy education programmes for patients and their families and carers should be continued into the treatment phase, with the aims of reviewing the original choice made by the patient, optimizing patient involvement in their own care, improving treatment adherence, and fostering good communication and collaborative relationships with caregivers (2C).
**Canadian Society of Nephrology (2014)**	-
**ERBP (2015)**	We recommend giving priority to the patient's general status and preference in selecting renal replacement therapy as there is an absence of evidence of superiority of one modality over another in patients with diabetes and CKD stage 5 (1C).
**National Kidney Foundation KDOQI (2015)**	-
**KHA – CARI (2015)**	We recommend that patients with end-stage kidney disease due to autosomal dominant polycystic kidney disease be considered for either hemodialysis or peritoneal dialysis where chronic dialysis is required (refer to KHA-CARI Guideline: Acceptance Onto Dialysis) (1C).
**Spain Ministry of Health (2016)**	-
**ERBP (2016)**	-
**UK Renal Association (2017)**	-
**NICE (2018)**	Ensure that decisions about RRT modalities or conservative management are made jointly with the person (or with their family members or carers for children or adults lacking capacity) and healthcare team, taking into account: predicted quality of life, predicted life expectancy and the person's preferences factors such as co-existing conditions.<br><br>Offer people (and their family members or carers, as appropriate) regular opportunities: to review the decision regarding RRT modalities or conservative management to discuss any concerns or changes in their preferences.<br><br>Offer a choice of dialysis modalities at home or in center ensuring that the decision is informed by clinical considerations and patient preferences.
**International Society of Peritoneal Dialysis (2021)**	The principles of person-centered care and shared decision-making should be applied to the care of people who are reaching end-stage kidney disease and this includes ensuring that they all have the opportunity to learn about peritoneal dialysis (PD) and to have a meaningful opportunity to be treated with it if that is their choice. It should be kept in mind by the PD care team that many of these patients are anxious, distressed, and disadvantaged and they should be supported accordingly (Level of Evidence – Not applicable)<br><br>PD should be prescribed using shared decision-making between the person doing PD/ their caregivers and the care team with the aim of achieving realistic care goals to maximize quality of life and satisfaction for the individual, minimize their symptoms and provide high quality care (practice point).

Clinical Practice Guideline	Theme 3: interventions to support the decision-making process about dialysis modality selection
**Chile Ministry of Health (2010)**	Patients with CKD stage 4 (GFRe <30ml/min/m2) must receive information about options of renal replacement treatment: HD, PD, KT (recommendation B)
**KDIGO (2013)**	A multidisciplinary team should include or have access to dietary counselling, education and counselling about different RRT modalities, transplant options, vascular access surgery, and ethical, psychological, and social care. (Not Graded)
**UK Renal Association (2013)**	We recommend that all patients with severe CKD (stage 5 and progressive stage 4), together with their families and carers, should be offered an appropriate education programme aimed at improving their knowledge and understanding of their condition, and to help them choose among the options for treatment (1B).<br><br>We suggest that education programmes should be tailored to the needs of the individual, be based on the principles of adult learning and be designed to support patient choice. A variety of approaches should be available to support patient choice. The information imparted should be relevant to the person, the stage of their disease and treatment options available to them with the method, scale, pace and scope of the delivery being suited to the individual's learning style, capacity and preferences. The programme should also include provision for the education of patients who present late, and initiate dialysis in an unplanned fashion (2C).<br><br>We recommend that patients who present with advanced kidney failure and are likely to need RRT within 3 months should be able to access an accelerated care pathway to deliver education, information and prepare for RRT (1D). Patients presenting late or starting dialysis without any prior nephrology input should receive similar education and information about renal replacement therapies to inform long term choices.<br><br>We recommend that patients with advanced kidney disease (CKD Stage 4 & 5) should be given an estimate of their prognosis and quality of life both with and without renal replacement therapy (1C)
**Canadian Society of Nephrology (2014)**	-
**ERBP (2015)**	We recommend providing patients with unbiased information about the different available treatment options (1A).
**National Kidney Foundation KDOQI (2015)**	Patients who reach CKD stage 4 (GFR 30 mL/min/1.73 m2), including those who have imminent need for maintenance dialysis at the time of initial assessment, should receive education about kidney failure and options for its treatment, including kidney transplantation, PD, HD in the home or in-center, and conservative treatment. Patients' family members and caregivers also should be educated about treatment choices for kidney failure. (Not Graded)
**KHA – CARI (2015)**	-
**Spain Ministry of Health (2016)**	In patients with end-stage CKD in which different options for dialysis, transplantation or conservative treatment are proposed, the use of some type of patient decision aid is recommended to help make shared decisions (STRONG).<br><br>In patients with CKD in which some type of patient decision aid is used to help make shared decisions, it is suggested that it be provided with sufficient time and that it addresses the impact of the proposed interventions (WEAK).
**ERBP (2016)**	We recommend that the 4-variable KFRE performs sufficiently well for use in older patients with advanced CKD and eGFR <45 mL/min/1.73 m2 (1B)<br><br>We suggest using the Bansal score to predict individual 5-year risk of death before ESKD in non-frail older patients with CKD stage 3–5 (2B).<br><br>We suggest that in patients at low risk in the Bansal score, a score including the assessment of frailty as stated in question 4a be performed (2B).<br><br>We suggest that the REIN score be used to predict the risk for mortality in older patients with CKD stage 5 (2B).<br><br>We recommend that the option for Conservative Management be discussed during the shared decision-making process on different management options for ESKD (1D).
**UK Renal Association (2017)**	We recommend that Peritoneal Dialysis should be delivered in the context of a comprehensive and integrated service for renal replacement therapies, including hemodialysis (including temporary backup facilities), transplantation and conservative care. Both continuous ambulatory peritoneal dialysis (CAPD) and automated peritoneal dialysis (APD), in all its forms should be available (1C).<br><br>We recommend that all patients (and parents of pediatric patients) should, where possible, be adequately prepared for renal replacement therapy and this should include receiving information and education about PD treatment, delivered by an experienced member of the MDT. Patients commencing RRT in an unplanned

	fashion for whatever reason should receive this information once appropriate (1C).<br><br>Fast track education and urgent PD catheter insertion with acute start of PD should be available and be offered to suitable patients urgently starting on RRT who wish to avoid temporary hemodialysis (1C).
**NICE (2018)**	To enable people, and their families and carers (as appropriate), to make informed decisions, offer balanced and accurate information about: all treatments available to them (including RRT modalities and conservative management), and how the treatments may affect their lives.<br><br>Recognize the psychological impact of a person being offered RRT or conservative management and discuss what psychological support may be available to help with decision-making.<br><br>Discuss with people which treatment options are available to them and explain why any options may be inappropriate or not advised.<br><br>Offer oral and written information and support early enough to allow time for people to fully understand their treatment options and make informed decisions. Information should be in an accessible format.<br><br>Direct people to other sources of information and support (for example, online resources, pre-dialysis classes and peer support).<br><br>Remember that some decisions must be made months before RRT is needed (for example, a fistula is created at least 6 months before starting dialysis).<br><br>Be prepared to discuss the information provided both before and after decisions are made, in line with the person's wishes.<br><br>Take into account information the person has obtained from other sources (such as family members and carers) and how this information has influenced their decision.<br><br>Ensure that healthcare professionals offering information have specialist knowledge about late stage chronic kidney disease and the skills to support shared decision-making (for example, presenting information in a form suitable for developmental stage).<br><br>Offer people who have presented late, or who started dialysis in an unplanned way, the same information as people who present at an earlier stage.
**International Society of Peritoneal Dialysis (2021)**	-
