# Supplementary material for: Recommendations of high-quality clinical practice guidelines related to the process of starting dialysis: A systematic review
Source: PLoS One. 2022 Jun 13;17(6):e0266202. doi: 10.1371/journal.pone.0266202 (PMC9191707; doi:10.1371/journal.pone.0266202)
Supplement: Attachment — Submitted filename: PONE-D-21-37316_rev.docx [file pone.0266202.s007.docx]

PLOS ONE

#### Recommendations of high-quality clinical practice guidelines related to the process of starting dialysis: a systematic review.

--Manuscript Draft--

| Manuscript Number: | PONE-D-21-37316 |
| --- | --- |
| Article Type: | Research Article |
| Full Title: | Recommendations of high-quality clinical practice guidelines related to the process of starting dialysis: a systematic review. |
| Short Title: | High-quality clinical practice guidelines recommendations related to the process of starting dialysis |
| Corresponding Author: | Karla Salas-Gama  Vall d'Hebron Hospital Universitari Barcelona, SPAIN |
| Keywords: | chronic kidney disease; clinical practice guidelines; dialysis; Systematic review |
| Abstract: | Background  The optimal time for initiation of dialysis and which modality to choose as the starting therapy is currently unclear. This systematic review aimed to assess the recommendations across high-quality clinical practice guidelines (CPGs) related to the start of dialysis.  Methods  We systematically searched MEDLINE, EMBASE, Web of Science, LILACS, and databases of organisations that develop CPGs between September 2008 to August 2021 for CPGs that addressed recommendations on the timing of initiation of dialysis, selection of dialysis modality, and interventions to support the decision-making process to select a dialysis modality. We used the Appraisal of Guidelines for Research and Evaluation instrument to assess the methodological quality of the CPGs and included only high-quality CPGs. This study is registered in PROSPERO, number CRD42018110325.  Results  We included 12 high-quality CPGs. Six CPGs addressed recommendations related to the timing of initiating dialysis, and all agreed on starting dialysis in the presence of symptoms or signs. Six CPGs addressed recommendations related to the selection of modality but varied greatly in their content. Nine CPGs addressed recommendations related to interventions to support the decision-making process. Eight CPGs agreed on recommended educational programs that include information about dialysis options.  One CPG considered using patient decision aids a strong recommendation. Limitations  We could have missed potentially relevant guidelines since we limited our search to CPGs published from 2008, and we set up a cut-off point of 60% in domains of the rigour of development and editorial independence.  Conclusion  High-quality CPGs related to the process of starting dialysis were consistent in initiating dialysis in the presence of symptoms or signs and offering patients education at the point of decision-making. There was variability in how CPGs addressed the issue of dialysis modality selection. CPGs should improve strategies on putting recommendations into practice and the quality of evidence to aid decision-making for patients.  Funding None |
| Order of Authors: | Karla Salas-Gama |
|  | Igho J. Onakpoya |
|  | Jorge Coronado Daza |
|  | Rafael Perera |
|  | Carl J. Heneghan |

| Additional Information: |  |
| --- | --- |
| Question | Response |
| Financial Disclosure  Enter a financial disclosure statement that describes the sources of funding for the work included in this submission. Review the [submission guidelines](http://journals.plos.org/plosone/s/submission-guidelines#loc-financial-disclosure-statement) for detailed requirements. View published research articles from [PLOS ONE](http://journals.plos.org/plosone/) for specific examples.  This statement is required for submission and will appear in the published article if the submission is accepted. Please make sure it is accurate. | The author(s) received no specific funding for this work. |
| Unfunded studies  Enter: The author(s) received no specific funding for this work.  Funded studies  Enter a statement with the following details:   - Initials of the authors who received each award - Grant numbers awarded to each author - The full name of each funder - URL of each funder website - Did the sponsors or funders play any role in the study design, data collection and analysis, decision to publish, or preparation of the manuscript? - NO - Include this sentence at the end of your statement: The funders had no role in study design, data collection and analysis, decision to publish, or preparation of the manuscript. - YES - Specify the role(s) played. |  |
| * typeset |  |
|  |  |

| This statement is required for submission and will appear in the published article if the submission is accepted. Please make sure it is accurate and that any funding sources listed in your Funding Information later in the submission form are also declared in your Financial Disclosure statement.  View published research articles from  [PLOS ONE](http://journals.plos.org/plosone/) for specific examples. | Foundation Trust). RP hold grant funding from the NIHR Programme of Applied Research. He leads a programme looking at how general practitioners manage chronic kidney disease and chronic heart failure.  KS and JC have no interests to declare. |
| --- | --- |
|  |  |
| * typeset |  |
| Ethics Statement  Enter an ethics statement for this submission. This statement is required if the study involved:   - Human participants - Human specimens or tissue - Vertebrate animals or cephalopods - Vertebrate embryos or tissues - Field research   Write "N/A" if the submission does not require an ethics statement.  General guidance is provided below. Consult the [submission guidelines](http://journals.plos.org/plosone/s/submission-guidelines#loc-guidelines-for-specific-study-types) for detailed instructions. Make sure that all information entered here is included in the | N/A |

| Methods section of the manuscript. |  |
| --- | --- |
| Format for specific study types  Human Subject Research (involving human participants and/or tissue)   - Give the name of the institutional review board or ethics committee that approved the study - Include the approval number and/or a statement indicating approval of this research - Indicate the form of consent obtained (written/oral) or the reason that consent was not obtained (e.g. the data were analyzed anonymously)   Animal Research (involving vertebrate animals, embryos or tissues)   - Provide the name of the Institutional Animal Care and Use Committee (IACUC) or other relevant ethics board that reviewed the study protocol, and indicate whether they approved this research or granted a formal waiver of ethical approval - Include an approval number if one was obtained - If the study involved non-human primates, add additional details about animal welfare and steps taken to ameliorate suffering - If anesthesia, euthanasia, or any kind of animal sacrifice is part of the study, include briefly which substances and/or methods were applied   Field Research  Include the following details if this study involves the collection of plant, animal, or other materials from a natural setting:   - Field permit number - Name of the institution or relevant body that granted permission |  |
|  |  |
| Data Availability  Authors are required to make all data underlying the findings described fully available, without restriction, and from the time of publication. PLOS allows rare exceptions to address legal and ethical concerns. See the [PLOS Data Policy](http://journals.plos.org/plosone/s/data-availability) and [FAQ](http://journals.plos.org/plosone/s/data-availability#loc-faqs-for-data-policy) for detailed information. | Yes - all data are fully available without restriction |

| A Data Availability Statement describing where the data can be found is required at submission. Your answers to this question constitute the Data Availability Statement and will be published in the article, if accepted. |  |
| --- | --- |
| Important: Stating ‘data available on request from the author’ is not sufficient. If your data are only available upon request, select ‘No’ for the first question and explain your exceptional situation in the text box. |  |
| Do the authors confirm that all data underlying the findings described in their manuscript are fully available without restriction? |  |
| Describe where the data may be found in full sentences. If you are copying our sample text, replace any instances of XXX with the appropriate details.   - If the data are held or will be held in a public repository, include URLs, accession numbers or DOIs. If this information will only be available after acceptance, indicate this by ticking the box below. For example: All XXX files are available from the XXX database (accession number(s) XXX, XXX.). - If the data are all contained within the manuscript and/or Supporting Information files, enter the following: All relevant data are within the manuscript and its Supporting Information files. - If neither of these applies but you are able to provide details of access elsewhere, with or without limitations, please do so. For example:   Data cannot be shared publicly because of [XXX]. Data are available from the XXX Institutional Data Access / Ethics Committee (contact via XXX) for researchers who meet the criteria for access to confidential data.  The data underlying the results | All relevant data are within the manuscript and its Supporting Information files. |

| presented in the study are available from (include the name of the third party and contact information or URL).   - This text is appropriate if the data are owned by a third party and authors do not have permission to share the data.   * typeset |  |
| --- | --- |
| Additional data availability information: |  |

Manuscript	[Click here to access/download;Manuscript;Manuscript.docx](https://www.editorialmanager.com/pone/download.aspx?id=30214035&guid=c46cf602-8a85-4d48-bc24-13f894834370&scheme=1)

# 1. ABSTRACT

## 1. Background

1. The optimal time for initiation of dialysis and which modality to choose as the starting
2. therapy is currently unclear. This systematic review aimed to assess the recommendations
3. across high-quality clinical practice guidelines (CPGs) related to the start of dialysis.

## 4. Methods

5. We systematically searched MEDLINE, EMBASE, Web of Science, LILACS, and databases
6. of organisations that develop CPGs between September 2008 to August 2021 for CPGs that
7. addressed recommendations on the timing of initiation of dialysis, selection of dialysis
1. modality, and interventions to support the decision-making process to select a dialysis
2. modality. We used the Appraisal of Guidelines for Research and Evaluation instrument to
3. assess the methodological quality of the CPGs and included only high-quality CPGs. This
4. study is registered in PROSPERO, number CRD42018110325.

## 1. Results

2. We included 12 high-quality CPGs. Six CPGs addressed recommendations related to the
3. timing of initiating dialysis, and all agreed on starting dialysis in the presence of symptoms or
4. signs. Six CPGs addressed recommendations related to the selection of modality but varied
5. greatly in their content. Nine CPGs addressed recommendations related to interventions to
6. support the decision-making process. Eight CPGs agreed on recommended educational
7. programs that include information about dialysis options. One CPG considered using patient
8. decision aids a strong recommendation.

## 9. Limitations

23. We could have missed potentially relevant guidelines since we limited our search to CPGs
24. published from 2008, and we set up a cut-off point of 60% in domains of the rigour of
25. development and editorial independence.

## 26. Conclusion

27. High-quality CPGs related to the process of starting dialysis were consistent in initiating
28. dialysis in the presence of symptoms or signs and offering patients education at the point of
29. decision-making. There was variability in how CPGs addressed the issue of dialysis modality
30. selection. CPGs should improve strategies on putting recommendations into practice and the
31. quality of evidence to aid decision-making for patients.

## 32. Funding

33. None

34

## 35. KEYWORDS

1. Chronic Kidney Disease, Clinical Practice Guidelines, Dialysis, Systematic Review

# 37. INTRODUCTION

38. Chronic kidney disease (CKD) is a global health problem with more than one in ten adults
39. affected [1]. Patients with end-stage kidney disease (ESKD) require renal replacement
40. therapy (RRT), and most will do it with one of the two dialysis modalities: peritoneal dialysis
41. (PD) or haemodialysis (HD). Starting dialysis is a complex decision, and the optimal time for
42. starting is unclear. The only randomised controlled trial that analysed an early vs late-start
43. showed no differences between the two approaches, [2] and concluded that with careful
44. clinical management, dialysis should be delayed until eGFR reaches 7ml/min/1.73m^2^ or
45. clinical symptoms are present.
46. There is currently insufficient evidence about which modality to choose as the starting
47. therapy. In 2018, more than three million people worldwide were on dialysis. Of these, only
48. 11% were on PD [3]. A randomised controlled trial [4] compared starting dialysis with HD vs
49. PD showed no differences in mortality at two years. The results suggested a hypothesis of
50. equivalence between PD and HD. A previous Cochrane review [5] also concluded there was
51. insufficient data to draw conclusions. Given the lack of good quality evidence to recommend
52. one modality over the other and considering that HD and PD have different practical factors,
53. including harms and benefits that will potentially impact a person’s life, it is particularly
54. important to offer patients an evidence-based, individualised decision-making process.
55. Clinical practice guidelines (CPGs) offer users clinical recommendations for daily practice
56. based on the best available evidence. The Institute of Medicine defines CPGs as
57. ‘recommendations for clinicians about the care of patients with specific conditions. They
58. should be based upon the best available research evidence and practice experience’ [6].
59. However, clinicians are faced with guidelines of variable quality and not sufficiently
60. transparent with respect to principles for guideline development, evidence review, or
61. potential conflicts of interest [7]. To be able to use CPGs in clinical practice, their quality
62. needs to be ensured.
63. Several CPGs for the management of CKD have been published in different countries.
64. However, no systematic assessment has been done on the process of starting dialysis
65. recommendations. The objective of this systematic review was to assess the consistency
66. across CPGs recommendations in three essential themes: timing of initiation of dialysis,
67. selection of dialysis modality, and the interventions to support the decision-making process
68. about dialysis modality selection.

# 69. METHODS

70. This study was reported in line with the Preferred Reporting Items for Systematic Reviews
71. and Meta-Analysis (PRISMA) statement [8]. No amendments were made to the protocol after
72. its registration other than an updated search.

## 73. Inclusion and exclusion criteria

74. **Population:** We included high-quality CPGs related to the process of starting dialysis in
75. adults (18 years or more), published in English between January 2008 until the present. When
76. the translation was possible, non-English CPGs were also included. Where more than one
77. version of the same guideline was found, we included only the most updated version. We
78. defined high-quality CPGs as guidelines that were evidence-based (explicitly describing how
79. the evidence was assessed) and that obtained a minimum score of 60% in domain 3 (Rigor of
80. Development) and domain 6 (Editorial Independence) at the Appraisal of Guidelines for
81. Research and Evaluation (AGREE II) instrument [9]. We considered these domains because
82. the rigour of guideline development and the editorial independence of authors seem to have
83. the strongest influence on the overall assessment of guideline quality and recommendation
84. for use [10]. We established the cut-off of 60% before beginning the AGREE II appraisals
85. based on values adopted by other authors [11-13].
86. **Interventions:** CPGs recommendations related to three specific themes of the process of
87. starting dialysis: a) timing of initiation of dialysis (criteria to when to start dialysis treatment),
88. b) dialysis modality selection (in-centre or home HD, or ambulatory or continuous automatic
89. PD), and c) interventions to support the decision-making process about dialysis modality
90. selection (any tool designed to help people participate in share-decision making process, like
91. educational programs, decision aids, algorithms, peer support programs, etc.).
92. **Outcomes:** a) Variation across the content of included high-quality CPG recommendations
93. on the process of initiating dialysis in CKD patients; b) methodological quality of included
94. guidelines.
95. We also collected data on organisation or author, year, country, language, target population,
96. level of evidence and grade of recommendation.
97. **Exclusion criteria:** CPGs that did not offer recommendations related to the timing of
98. initiation of dialysis treatment, dialysis modality selection, or interventions to support the
99. decision-making process were excluded. CPGs focused on acute kidney failure, pregnancy, or
1. paediatric populations were excluded. Adaptations, translations, commentaries, or summaries
2. were also excluded.

## 1. Identification of clinical practice guidelines

2. CPGs were identified through a systematic search of MEDLINE, Web of Science, EMBASE,
3. LILACS, and databases of organisations that develop CPGs like the National Institute for
4. Health and Care Excellence (NICE), Scottish Intercollegiate Guidelines, and the Guidelines
5. International Network. We supplemented this by searching societies that perform research
107. related to CKD. We performed a systematic search on the 14^th^ September 2018, combining
108. Medical Subject Headings (MeSH) terms and words related to CKD, ESKD, dialysis and
109. RRT, limiting the results to Practice Guideline [publication type]. A full search strategy is
110. shown in S1 Appendix. We updated the database search on 2^nd^ August 2021. We used the
111. same search method, except that we narrowed the searches to 2018 onwards.

## 112. Selection of high-quality CPGs and data extraction

113. One reviewer (KS) screened the titles and abstracts of all records and discarded those that
114. were duplicates or that were not pertinent for the study. Two reviewers (JC and KS)
115. independently assessed the full text of potentially relevant guidelines and selected those that
116. met the inclusion criteria. Disagreements were resolved through discussion. When consensus
117. could not be reached, a third reviewer (IJO) was consulted.
118. We used the AGREE II instrument to identify high-quality CPGs from the previously
119. selected guidelines [9]. The AGREE II instrument contains 23 items distributed along six
120. domains of guideline development: Scope and Purpose, Stakeholder Involvement, Rigor of
121. Development, Clarity of Presentation, Applicability, and Editorial Independence; and two
122. overall assessments. Each item is rated on a seven-point Likert scale from 1 (strongly
123. disagree) to 7 (strongly agree). A quality domain score between 0% and 100% is calculated
124. based on this rating for each of the six domains [9].
125. Once high-quality CPGs were identified, one reviewer (KS) extracted the text, quality of
126. evidence and strength of recommendations. This information was independently verified by
127. the second reviewer (IJO). We did not assess the quality of the underlying evidence. Two
128. reviewers assessed the content and variation across recommendations. The first reviewer
129. (KS) identified topics covered by the recommendations and codified them. Codes then were
130. reassessed through a comparison of each CPG recommendation to identify similarities and
131. discrepancies. Finally, recommendations were grouped along the topics to analyse variation
132. across CPGs. A second reviewer (IJO) then verified this information independently.

## 133. Statistical analysis

134. We analysed the data using descriptive statistics: absolute and relative frequencies for
135. categorical variables, measures of central tendency and dispersion for continuous variables. A
136. quality score was calculated for each of the six AGREE II domains by summing up all the
137. scores of the individual items in a domain and by scaling the total as a percentage of the
138. maximum possible score for that domain [9]. The intraclass correlation coefficient (ICC) was
139. used to measure inter-rater reliability in the AGREE II instrument scores. The degree of
140. reliability was measured using the following ICC definitions: ≤0.50 poor reliability; 0.51-
141. 0.75 moderate reliability; 0.76-0.90 good reliability; greater than 0.90 excellent reliability
142. [14]. We conducted a narrative, descriptive synthesis to analyse the consistency between
143. CPGs recommendations.

# 1. RESULTS

## 2. Search and characteristics of included guidelines

3. The first main search retrieved a total of 2628 records, of which 1040 were duplicates. We
4. excluded 1481 documents at the title and abstract screening, leaving 107 eligible records for
5. full-text analysis. Eighty-nine records were excluded because they did not meet the inclusion
6. criteria. The remaining 18 CPGs were assessed with the AGREE-II instrument. Seven were
7. excluded because they did not obtain a minimum score of 60% in domains 3 (Rigour of
8. development) or 6 (Editorial independence). References and reasons for the exclusion of
9. CPGs excluded in this phase are given in S2 Table. This left 11 high-quality CPGs for
10. inclusion [15-25]. A PRISMA flow diagram of the selection process is shown in Fig 1.

### 154. Fig 1. PRISMA Flow diagram

155. The updated searches in August 2021 resulted in a total of 712 records being screened and
156. assessed for eligibility. Four CPGs were evaluated with the AGREE-II instrument, and three
157. were excluded because they did not obtain a minimum score of 60% in domains 3 (Rigour of
158. development) or 6 (Editorial independence). References and reasons for the exclusion of
159. CPGs excluded in this phase are given in S2 Table. One new high-quality CPG was included
160. [26]. This selection process is also shown in Fig 1. In total, we screened 2101 records which
161. resulted in 12 high-quality CPGs being included in the review [15-26].
162. Table 1 presents the characteristics of the included CPGs. Half of them were developed in
163. Europe. Five CPGs (42%) were published within the last five years, and 10 (83%) were
164. published in English. Three CPGs (25%) were focused on a specific population: people with
165. diabetes mellitus and CKD [19], autosomal dominant polycystic kidney disease (ADPKD)
166. [21], and older adults (>65 years old) with CKD [23]. The level of evidence and strength of
167. recommendations was assessed through the Grades of Recommendation, Assessment,
168. Development and Evaluation (GRADE) approach in 11 (92%) of the included CPGs.
169. However, nine (75%) used a modified GRADE version. The remaining CPG (8%) used a
170. generic grading system. Table 2 shows the differences between the systems of quality of
171. evidence and strength of recommendation.
1. 172
173. **Table 1: Characteristics of included high-quality clinical practice guidelines.**

**Organisation	Name	Country (Year)**

**Language	Target population	Theme (s) included	Levels of evidence**

**Grade of recommendation**

**Chile Ministry of Health**	Clinical guideline: peritoneal dialysis	Chile

(2010)

Spanish	People with CKD treated

with PD

Theme 2 & 3	Unspecified*

KDIGO 2012 CPG for the Evaluation and Management of CKD

**KDIGO**

USA (2013)

English	People with CKD who are

not on RRT

Theme 1 & 3	Modified GRADE system

**UK Renal Association**	Planning, Initiating and Withdrawal of RRT	UK (2013)

English	People with CKD with established renal failure

Theme 1, 2 & 3	Modified GRADE system

**Canadian Society of**	CPG for timing the initiation of chronic dialysis

**Nephrology**

Canada	English (2014)

People with CKD for whom initiation of elective dialysis is planned

Theme 1	Standard GRADE system

CPG on the management of patients with diabetes and CKD stage 3b or higher

**ERBP**

Europe (2015)

English	People with DM and CKD

3b or higher

Theme 1, 2 & 3	Modified GRADE system

**National Kidney Foundation KDOQI**

KDOQI Clinical Practice Guideline for Haemodialysis Adequacy: 2015 update

US	English

(2015)

People with CKD treated with, initiating, or planning to initiate maintenance HD

Theme 1 & 3	Modified GRADE system

**KHA-CARI**	ADPKD Guideline: Management of End-Stage Kidney

Disease

Australia (2015)

English	People with ADPKD	Theme 2	Modified GRADE system

**Spain Ministry of Health**	CPG on detection and management of CKD	Spain

(2016)

Spanish	People with CKD who are

not on RRT

Theme 3	Standard GRADE system

CPG on the management of older patients with CKD stage 3b or higher

**ERBP**

Europe (2016)

English	Older people (>65 years)

with CKD

Theme 3	Modified GRADE system

**UK Renal Association**	CPG Peritoneal Dialysis in Adults and Children	UK

(2017)

English	People with CKD treated

with PD

Theme 3	Modified GRADE system

Renal replacement therapy and conservative management

**NICE**

UK (2018)

English	People with CKD 4 or 5	Theme 1, 2 & 3	Modified GRADE system

**International Society of Peritoneal Dialysis**

Prescribing High Quality Goal-Directed Peritoneal Dialysis

International (2020)

English	People with CKD treated

with PD

Theme 2	Modified GRADE system

| 174 | *Based on the quality of included studies and consensus amongst authors. |
| --- | --- |
| 175 | CKD: chronic kidney disease. PD: peritoneal dialysis. HD: haemodialysis. CPG: Clinical Practice Guideline. KDIGO: Kidney Disease: Improving Global Outcomes. GRADE: Grading of Recommendations |
| 176 | Assessment, Development and Evaluation. ERBP: European Renal Best Practice. KDOQI: Kidney Disease Outcomes Quality Initiative. KHA-CARI: Kidney Health Australia - Caring for Australasians with Renal |
| 177 | Impairment. NICE: National Institute for Health and Care Excellence. CEBM: Centre for Evidence-Based Medicine at Oxford. Theme 1: timing of initiating dialysis. Theme 2: selection of dialysis modality. Theme 3: |
| 178 | Interventions to support the decision-making process |
| 179 |  |

180	**Table 2: Levels of evidence and strength of re**

| **ORGANISATION** | **LEVELS OF EVIDENCE** | **STRENGTH OF RECOMMENDATIONS** |
| --- | --- | --- |
| **Chile Ministry of Health** | **1** = Randomized controlled trial  **2** = Cohort, Case-control, no randomised trial | **A** = Highly recommended, based in good-quality studies.  **B** = Based in moderate-quality studies. |
|  | **3** = Descriptive studies | **C** = Exclusively based in experts’ opinion or low-quality studies. |
|  | **4** = Experts' opinion | **I** = Insufficient information to give a recommendation |
| **Canadian Society of** | **Standard GRADE system** | **Standard GRADE system** |
| **Nephrology** | **High** = We are very confident that the true effect lies close to that of the estimate of the effect | **Strong** = Most individuals in this situation would want the recommended course of action and only a |
|  | **Moderate** = We are moderately confident in the effect estimate: The true effect is likely to be close to | small proportion would not. Most patients should receive the recommended course of action. |
| **Spain Ministry of Health** | the estimate of the effect, but there is a possibility that it is substantially different  **Low** = Our confidence in the effect estimate is limited: The true effect may be substantially different | **Weak** = The majority of individuals in this situation would want the suggested course of action, but many  would not. Different choices will be appropriate for different patients. Each patient needs help to arrive |
|  | from the estimate of the effect | at a management decision consistent with her or his values and preferences |
|  | **Very low** = We have very little confidence in the effect estimate: The estimate of effect is very |  |
|  | uncertain, and often will be far from the truth |  |
| **KDIGO** | **Modified GRADE system A**=High | **Modified GRADE system**  **Level 1** = 'We recommend' (use the same implication as strong recommendation of the standard GRADE |
| **ERBP** | **B**=Moderate  **C**=Low | system)  **Level 2** = 'We suggest' (use the same implication as weak recommendation of the standard GRADE |
| **KDOQI** | **D**=Very low | system ) |
|  |  | **Not Graded** / **Practice Point** = Used to provide guidance based on common sense or where the topic |
| **KHA – CARI** | (same meaning as the standard GRADE system for the four categories) | does not allow adequate application of evidence. The ungraded recommendations are generally written as  simple declarative statements but are not meant to be interpreted as being stronger recommendations than |
| **International Society** |  | Level 1 or 2 recommendations. |
| **of Peritoneal Dialysis** |  |  |
| **UK Renal Association** | **Modified GRADE system**  **Grade A** = High-quality evidence that comes from consistent results from well-performed randomised | **Modified GRADE system**  **Grade 1** = 'We recommend' (strong recommendation) |
|  | controlled trials, or overwhelming evidence of some other sort such as well-executed observational | **Grade 2** = 'We suggest' (weak recommendation) |
|  | studies with very strong effects | Use wording to indicate the strength of each recommendation. When making a strong recommendation |
|  | **Grade B** = Moderate-quality evidence from randomised trials that suffer from serious flaws in | guideline authors are encouraged to use ‘We recommend...’ and when making a weak recommendation |
|  | conduct, inconsistency, indirectness, imprecise estimates, reporting bias, or some combination of these | authors should use ‘We suggest…’ |
|  | limitations, or from other study designs with special strength |  |
|  | **Grade C** = Low-quality evidence from observational studies, or from controlled trials with several |  |
|  | very serious limitations |  |
|  | **Grade D** = Based only on case studies or expert opinion |  |
| **NICE** | **Modified GRADE system**  **High** = further research is very unlikely to change our confidence in the estimate of effect | **Modified GRADE system**  Use the wording of the recommendations: |
|  | **Moderate** = further research is likely to have an important impact on our confidence in the estimate of | Recommendations that should (or not should) be used = 'Offer' (or 'do not offer'), 'Advise', 'Ask about' or |
|  | effect and may change the estimate | 'Commission' |
|  | **Low** = further research is very likely to have an important impact on our confidence in the estimate of | Recommendation that could be used = 'Consider', 'Be aware of', 'Explore', 'Assess' or 'Think about' |
|  | effect and is likely to change the estimate | Recommendations that must (or must not) be used = 'Must' (or 'must not') |
|  | **Very Low** = any estimate of effect is very uncertain |  |

181

## 182. Methodological quality

1. Table 3 presents AGREE II domain scores for each high-quality CPG. The median scores and
2. the range for the domains were: scope and purpose 93% (72-100%); stakeholder involvement
3. 80% (39-100%); rigour of development 82% (60-100%); clarity and presentation 99% (97-
4. 100%); applicability 57% (17-96%); and editorial independence 88% (63-100%). The
5. intraclass coefficient (ICC) showed high values for all the CPGs, indicating good to excellent
6. reliability.
1. 189
190. **Table 3: Domain scores of high-quality CPGs according to the AGREE II instrument**

| **Scope and**  **Purpose** | **Stakeholder**  **involvement** | **Rigour of**  **development** | **Clarity and**  **presentation** | **Applicability** | **Editorial**  **independence** | **coefficient**  **(95% CI)** |
| --- | --- | --- | --- | --- | --- | --- |
| Chile Ministry of Clinical guideline: peritoneal dialysis 97 | 58 | 68 | 100 | 71 | 96 | 0.94 (0.71-0.99) |
| KDIGO KDIGO 2012 CPG for the Evaluation and _100_ | 92 | 90 | 100 | 46 | 92 | 0.98 (0.92-0.99) |
| UK Renal Planning, Initiating and Withdrawal of Renal _72_ | 42 | 60 | 97 | 44 | 63 | 0.88 (0.46-0.98) |
| Canadian Society of CPG for timing the initiation of chronic _100_ | 75 | 89 | 100 | 96 | 92 | 0.97 (0.85-0.99) |
| ERBP CPG on the management of patients with _100_ | 100 | 100 | 100 | 58 | 100 | 0.95 (0.73-0.99) |
| National Kidney KDOQI CPG for Haemodialysis Adequacy: _86_ | 39 | 82 | 97 | 17 | 71 | 0.96 (0.81-0.99) |
| KHA - CARI Autosomal Dominant Polycystic Kidney _81_ | 89 | 77 | 100 | 35 | 83 | 0.89 (0.50-0.98) |
| Spain Ministry of CPG on detection and management of CKD 100 | 97 | 97 | 100 | 81 | 92 | 0.99 (0.97-0.99) |
| ERBP CPG on the management of older patients _100_ | 94 | 100 | 100 | 58 | 96 | 0.99 (0.98-0.99) |
| UK Renal CPG Peritoneal Dialysis in Adults and _81_ | 78 | 60 | 97 | 38 | 79 | 0.76 (0.11-0.96) |
| NICE Renal replacement therapy and conservative _97_ | 94 | 96 | 100 | 96 | 96 | 0.99 (0.98-0.99) |
| International Society Prescribing High Quality Goal-Directed _100_ | 97 | 64 | 100 | 42 | 92 | 0.80 (0.20 – 0.97) |
| **Median scores (range)** 93 (72-100) | 80 (39-100) | 82 (60-100) | 99 (97-100) | 57 (17-96) | 88 (63-100) |  |

**Organization	Name**

**Domain scores (%)	Intraclass**

**correlation**

Health

Management of CKD

Association	Replacement Therapy

Nephrology	dialysis

diabetes and CKD stage 3b or higher

Foundation KDOQI	2015 update

Disease Guideline: Management of ESKD

Health

with CKD stage 3b or higher

Association	Children

management

of Peritoneal Dialysis	Peritoneal Dialysis

191. 191
192. 192

CPG: clinical practice guideline. KDIGO: Kidney Disease: Improving Global Outcomes. CKD: chronic kidney disease. ERBP: European Renal Best Practice. KDOQI: Kidney Disease Outcomes Quality Initiative. KHA- CARI: Kidney Health Australia - Caring for Australasians with Renal Impairment. ESKD: end-stage kidney disease. NICE: National Institute for Health and Care Excellence.

### 193. Variation across CPGs recommendations

194. While assessing and comparing the CPGs recommendations, we considered that CPGs had
195. different target populations and primary objectives and did not cover all the aspects analysed
196. in our systematic review (Table 1 and S3 Table). Table 4 shows the coded categories
197. extracted from the text of high-quality CPGs recommendations. S4 Table shows the complete
198. text of the recommendations.
199. **Table 4: High-quality CPGs recommendations related to the process of starting dialysis**

|  | **HIGH-QUALITY CPGs INCLUDED IN THE STUDY** | | | | | | | | | | | |
| --- | --- | --- | --- | --- | --- | --- | --- | --- | --- | --- | --- | --- |
| **CPGs RECOMMENDATIONS** | **1 (15)** | **2 (16)** | **3 (17)** | **4 (18)** | **5 (19)** | **6 (20)** | **7 (21)** | **8 (22)** | **9 (23)** | **10 (24)** | **11 (25)** | **12 (26)** |
| **TIMING OF DIALISYS INITIATION** | | | | | | | | | | | | |
| **With the onset of signs or symptoms** |  |  |  |  |  | **NG** |  |  |  |  |  |  |
| **Specific GFR (if symptoms are not present)** |  |  |  |  |  |  |  |  |  |  |  |  |
| **The decision to start should be based on joint discussion with the patient** |  |  |  |  |  |  |  |  |  |  |  |  |
| **SELECTION OF DIALYSIS MODALITY** | | | | | | | | | | | | |
| **Modality should be prescribed using shared decision making between person and care team** |  |  |  |  |  |  |  |  |  |  |  | **NG** |
| **Offer modalities and ensure that the decisions are informed** |  |  |  |  |  |  |  |  |  |  |  |  |
| **No superiority within dialysis modalities for persons with ADPKD and DM** |  |  |  |  |  |  |  |  |  |  |  |  |
| **Encourage PD and home-dialysis use** |  |  |  |  |  |  |  |  |  |  |  | **NG** |
| **Offer regular opportunities to review the original decision** |  |  |  |  |  |  |  |  |  |  |  |  |
| **INTERVENTIONS TO SUPPORT THE DECISION-MAKING PROCESS ABOUT DIALYSIS MODALITY SELECTION** | | | | | | | | | | | | |
| **Educational programs with information or counselling about RRT** |  | **NG** | ****** |  |  | **NG** |  |  |  |  | ****** |  |
| **Use of tools to predict CKD progression, prognosis, QoL, or mortality** |  |  |  |  |  |  |  |  |  |  |  |  |
| **Use of some type of patient decision-aid** |  |  |  |  |  |  |  |  |  |  | ***** | ***** |

200. NG = not graded. *Did not make a specific recommendation but offer information about this topic. **Include recommendations about characteristics of the provided information and skills of the healthcare professional that provides it.

1. **Quality of evidence**

202. 202

**Strength of recommendation**

Strong Weak

High

Moderate

Low

Very low

203. Six of the included CPGs (50%) addressed recommendations related to the timing of
204. initiating dialysis. We identified three topics across CPGs recommendations: 1) starting
205. dialysis in the presence of symptoms or signs related to CKD, 2) initiating dialysis at a
206. specific starting point (eGFR) in the absence of symptoms, and 3) ensuring that the decision
207. to start RRT is made jointly by the person and the healthcare team after a careful discussion.
208. All six CPGs agreed on starting dialysis in the presence of symptoms or signs. Three CPGs
209. considered this statement a strong recommendation, two CPGs considered it weak, and one
210. CPG did not grade the recommendation. The quality of evidence reported varied from very
211. low to high. Only two of the six CPGs recommended a specific eGFR of 5-7 ml/min/m^2^ as a
212. starting point in asymptomatic patients. The strength of the recommendation considered by

213. this two CPGs differed from one to another. Likewise, only two CPGs strongly recommended
214. that the decision to start dialysis should be made jointly between the patient and their
215. healthcare team (Table 4 and S4 Table).
216. Six of the included CPGs (50%) contained recommendations related to the selection of
217. dialysis modality. We identified five different topics across guidelines making it difficult to
218. assess the consistency across recommendations. Two CPGs explicitly mentioned the
219. principle of shared decision-making and recommended ensuring a joint decision. One CPG

220. made a strong recommendation, whereas the other one made a practice point. Two CPGs
221. made strong recommendations about offering all dialysis modalities, ensuring informed
222. decisions. Two CPGs with specific target populations (DM and ADPKD) strongly
223. recommended that either treatments, HD or PD, be considered since there is an absence of
224. evidence of the superiority of one modality over another for these populations. Three CPGs
225. made recommendations about encouraging the use of home dialysis (including PD) where
226. possible. Two CPGs made a weak recommendation, whereas the other one did not grade it.
227. Two CPGs recommended offering regular opportunities to review the original decision. One
228. CPG made a weak recommendation, whereas the other made a strong one (Table 4 and S4
229. Table).
230. Nine of included CPGs (75%) had recommendations related to interventions to support the
231. decision-making process about dialysis modality selection. Eight CPGs agreed to recommend
232. educational programs that contain information about the different RRT options. Almost all
233. CPGs considered this statement a strong recommendation. Two CPGs strongly recommended
234. using tools to predict clinical outcomes to guide clinicians to find the best RRT option. Only
235. one CPG considered using patient decision aids a strong recommendation based on moderate
236. quality of evidence. (Table 4 and S4 Table).

# 1. DISCUSSION

## 1. Summary of main findings

2. This systematic review of high-quality CPGs recommendations addressing the process of
3. starting dialysis showed that, overall, there is general consistency in initiating dialysis late in
4. the presence of symptoms or signs and offering patients education and information in the
5. moment of decision-making. Nevertheless, there is variability in how high-quality CPG

6. addresses the issue of dialysis modality selection and the use of other decision tools different
7. from education.
8. Across all CPGs, median domain scores at the AGREE II instrument were high, except for
9. domain 5 (Applicability), where only four CPGs scored over 60%. This finding is consistent
10. with what has already been described in studies that appraised the quality of CKD guidelines

248	[27-30].

249. Few CPGs addressed when to start dialysis in asymptomatic patients. Although the
250. recommended eGFR as the starting point was similar, the strength of the recommendation
251. differed. This uncertainty is also seen in the current clinical practice, where there is
252. variability in mean pre-dialysis eGFR among countries since a specific eGFR value for
253. initiating dialysis without symptoms has not been established [31]. Using only symptom-
254. based criteria might risk asymptomatic patients or those with subtle symptoms since there
255. may be difficulties in identifying them.
256. Although we found consistency in content and strength of recommendation related to
257. ensuring that the decision to start RRT is made jointly by the patient and the healthcare team,
258. we think this topic is still rarely tackled. Planning starting dialysis often includes
259. individualised discussions regarding patient values and preferences. With the increasing
260. recognition of the importance of person-centred care, it could be expected that more high-
261. quality CPGs recommended a joint discussion with the patient about the decision to start
262. dialysis. In this same way, shared-decision making is still a field that needs further discussion

263. along high-quality CPGs since only two stated a specific recommendation about it. There is
264. currently a need to provide more individualised care that incorporates patient goals and
265. preferences. Shared decision-making is a process during which patients, caregivers and
266. healthcare professionals collaborate to make healthcare choices. It depends on knowing and
267. understanding the best available evidence about the risks and benefits across all available
268. options while ensuring that the patient's values and preferences are considered [32]. The
269. initiative Choosing Wisely published in 2012 a recommendation that dialysis should not be
270. initiated without ensuring a shared decision-making process among patients, their families,
271. and the healthcare team [33].
272. A variety of interventions have been designed to help shared decision making to be
273. implemented into clinical practice: 1) interventions targeting healthcare professionals; 2)
274. interventions targeting patients; and 3) interventions targeting both [31]. In our systematic
275. review, only the CPG that focused on older people with CKD acknowledged

276. recommendations directed to healthcare professionals, advising the use of prediction models
277. or scores to predict progression or mortality in this population [23].
278. On the other hand, there was general consistency in high-quality CPGs in offering
279. interventions targeting patients. Most of the guidelines recommended offering educational
280. programs with information about the different RRT options. Few CPGs researched
281. recommend the use of patient decision aids. The Spain Ministry of Health’s CPG made a
282. strong recommendation based on moderate evidence for using decision aids to help CKD
283. patients make shared decisions [22]. In contrast, the NICE CPG committee was unable to
284. recommend that decision aids should be used because of the absence of evidence showing
285. clinically important benefits [25]. The International Society of Peritoneal Dialysis guideline
286. did not make a specific recommendation on this issue. Still, in the discussion section, they
287. suggest that decision aids should be provided, including audio-visual as well as written
288. material [26]. There is currently an increasing interest in using patient decision aids to
289. support patients with CKD to make treatment modality decisions. There is some ongoing
290. research that will offer more information about this topic [34, 35]. We did not find any high-
291. quality CPGs that included recommendations about interventions targeting both patients and
292. healthcare professionals.

## 1. Quality of evidence and strength of recommendations

2. We observed considerable variations in the quality of evidence and the strength of
3. recommendations across the CPGs that could be confusing since the implications of strong or
4. weak recommendations are highly different [36]. Possible explanations about these
5. differences have been previously described and included the year of CPG development, date
298. of search by guideline development groups, differences in the methods used to identify and
299. appraise evidence, or differences in the interpretation of the evidence [37]. Most of the CPGs
300. included in our systematic review used a modified GRADE approach. Since GRADE aims to
301. reduce confusion arising from multiple systems for grading evidence and recommendations,
302. it would be reasonable to adopt the standard GRADE system. This could facilitate the
303. comparison and understanding of the terminology.

## 304. Comparison with the existing literature

305. Although we did not identify systematic reviews of CPGs addressing starting dialysis, we
306. found a meta-synthesis of qualitative studies aimed to understand the process of decision-
307. making in persons with CKD [38]. The review found that modality decisions are highly
308. personal and strongly influenced by personal values. There is a need for planned and timely
309. discussions about modalities in which home-based dialysis is presented as a viable option.

## 310. Strength and limitations

311. We conducted comprehensive searches to identify relevant CPGs that addressed
312. recommendations about the process of starting dialysis. We used a two-step process to
313. identify high-quality CPGs. We used a validated tool (AGREE II) to assess the quality of
314. included guidelines independently, and we obtained high ICC values meaning good
315. reliability.
316. However, we recognise some limitations. We could have missed potentially relevant
317. guidelines since we limited our search to CPGs published from 2008, and we set up a cut-off
318. point of 60% in domains of the rigour of development and editorial independence for
319. defining high-quality CPGs. Although we used the AGREE II instrument to assess the
320. methodological quality of the CPGs, we did not evaluate the evidence underlying the

321. recommendations.

## 322. Implications for research

323. There is a dearth of evidence to inform current guidelines on when to start dialysis in
324. asymptomatic patients and which modality to choose. We found variability in how CPGs
325. reported the reasons and judgments behind the recommendations, suggesting there is
326. variation in how CPGs panels interpret and appraise the evidence. CPGs panels could
327. consider adopting the GRADE Evidence to Decision framework [39, 40] for a structured
328. approach in developing recommendations. This framework would facilitate the report of
329. reasons and judgment that determine the direction and strength of recommendations across
330. the different CPGs and allow comparison of recommendations from different CPGs panels.

## 331. Implications for clinical practice

332. Since initiating and selecting a modality has as a centrepiece an individualised shared
333. decision, offering individuals information about RRT options is probably not enough. High-
334. quality CPGs should emphasise the best strategies and interventions to assess and incorporate

335. people’s values and preferences into clinical practice in their dialysis modality selection.

# 1. CONCLUSIONS

2. High-quality CPGs related to the process of starting dialysis were consistent in initiating
3. dialysis late, in the presence of symptoms or signs, and offering patients education and
4. information in the moment of decision-making. There was, however, variability in how high-
5. quality CPG addresses the issue of dialysis modality selection and the use of other decision
6. tools different from education. There is variation in the process used by different CPGs
342. panels to appraise the quality of evidence and grade the strength of recommendation. CPGs
343. should improve their strategy on putting recommendations into practice and the quality of
344. evidence to aid patients' decision-making.

## 345. REGISTRATION

346. The protocol of this systematic review has been registered in the international prospective
347. register of systematic reviews (PROSPERO) under the registration number: CRD

348	CRD42018110325.

## 349. ACKNOWLEDGEMENTS

1. Karla Salas-Gama is a PhD candidate at the Methodology of Biomedical Research and Public
2. Health program, Universitat Autònoma de Barcelona, Spain.

## 3. LIST OF ABBREVIATIONS

4. AGREE II: Appraisal of Guidelines for Research and Evaluation - II
5. CKD: chronic kidney disease

6. CPGs: clinical practice guidelines
7. eGFR: estimated glomerular filtration rate
8. ESKD: end-stage kidney disease
9. ADPKD: autosomal dominant polycystic kidney disease
10. GRADE: Grades of Recommendation, Assessment, Development and Evaluation
11. HD: haemodialysis
361. NICE: National Institute for Health and Care Excellence
362. PD: peritoneal dialysis
363. PRISMA: Preferred Reporting Items for Systematic Reviews and Meta-Analysis
364. RRT: renal replacement therapy

# 365. References

1. 1. Hill NR, Fatoba ST, Oke JL, Hirst JA, O'Callaghan CA, Lasserson DS, Hobbs FD.
1. Global Prevalence of Chronic Kidney Disease - A Systematic Review and Meta-

368	Analysis. PLoS One. 2016;11(7):e0158765.

369. 2. Cooper BA, Branley P, Bulfone L, Collins JF, Craig JC, Fraenkel MB, Harris A,
1. Johnson DW, Kesselhut J, Li JJ, Luxton G, Pilmore A, Tiller DJ, Harris DC, Pollock
2. CA; IDEAL Study. A randomised, controlled trial of early versus late initiation of

372	dialysis. N Engl J Med. 2010;363(7):609-19.

373. 3. Fresenius Medical Care. Fresenius Medical Care 2018 Annual Report 2018; 2–266.
374. 4. Korevaar JC, Feith GW, Dekker FW, van Manen JG, Boeschoten EW, Bossuyt PM,
1. Krediet RT; NECOSAD Study Group. Effect of starting with hemodialysis compared
2. with peritoneal dialysis in patients new on dialysis treatment: a randomised controlled

377	trial. Kidney Int. 2003;64(6):2222-8.

378. 5. Vale L, Cody J, Wallace S, Daly C, Campbell M, Grant A, Khan I, Donaldson C,
1. Macleod A. Continuous ambulatory peritoneal dialysis (CAPD) versus hospital or
2. home haemodialysis for end-stage renal disease in adults. Cochrane Database Syst

381	Rev. 2004;(4):CD003963.

382. 6. IOM (Institute of Medicine). Clinical Practice Guidelines We Can Trust. Washington,
1. DC: The National Academies Press. 2011.
2. 7. Incze M, Ross JS. On the Need for (Only) High-Quality Clinical Practice Guidelines.

385	JAMA Intern Med. 2019;179(4):561.

386. 8. Page MJ, Moher D, Bossuyt PM, et al. PRISMA 2020 explanation and elaboration:
1. updated guidance and exemplars for reporting systematic reviews. BMJ.

388	2021;372:n160.

389. 9. Brouwers MC, Kho ME, Browman GP, Burgers JS, Cluzeau F, Feder G, Fervers B,
1. Graham ID, Grimshaw J, Hanna SE, Littlejohns P, Makarski J, Zitzelsberger L, for
2. the AGREE Next Steps Consortium. AGREE II: Advancing guideline development,
3. reporting and evaluation in healthcare. CMAJ 2010;182:E839-842.
4. 10. Hoffmann-Eßer W, Siering U, Neugebauer EAM, Brockhaus AC, McGauran N,
5. Eikermann M. Guideline appraisal with AGREE II: online survey of the potential
6. influence of AGREE II items on overall assessment of guideline quality and
7. recommendation for use. BMC Health Serv Res. 2018;18(1):143.
8. 11. Hoffmann-Eßer W, Siering U, Neugebauer EAM, Lampert U, Eikermann M.
9. Systematic review of current guideline appraisals performed with the Appraisal of
10. Guidelines for Research & Evaluation II instrument-a third of AGREE II users apply
11. a cut-off for guideline quality. J Clin Epidemiol. 2018;95:120-127.
12. 12. Molino CGRC, Leite-Santos NC, Gabriel FC, et al. Factors Associated With High-
13. Quality Guidelines for the Pharmacologic Management of Chronic Diseases in
14. Primary Care: A Systematic Review. JAMA Intern Med. 2019;179(4):553-560.
15. 13. Arevalo-Rodriguez I, Pedraza OL, Rodríguez A, et al. Alzheimer’s disease dementia
16. guidelines for diagnostic testing: a systematic review. Am J Alzheimers Dis Other

406	Demen. 2013;28(2):111-119.

407. 14. Koo TK, Li MY. A Guideline of Selecting and Reporting Intraclass Correlation
1. Coefficients for Reliability Research. J Chiropr Med. 2016;15(2):155-63.
2. 15. Ministerio de salud. Guía clínica diálisis peritoneal. Santiago: MINSAL, 2010.
3. 16. Kidney Disease: Improving Global Outcomes (KDIGO) CKD Work Group. KDIGO
4. 2012 clinical practice guideline for the evaluation and management of chronic kidney
5. disease. Kidney Int Suppl. 2013;3:1-150.
413. 17. Farrington K, Warwick G. Renal Association Clinical Practice Guideline on planning,
414. initiating and withdrawal of renal replacement therapy. Nephron Clin Pract. 2011;118

415	Suppl 1:c189-208.

416. 18. Nesrallah GE, Mustafa RA, Clark WF, et al; Canadian Society of Nephrology.
1. Canadian Society of Nephrology 2014 clinical practice guideline for timing the
2. initiation of chronic dialysis. CMAJ. 2014;186(2):112-7.
3. 19. Guideline development group. Clinical Practice Guideline on management of patients
4. with diabetes and chronic kidney disease stage 3b or higher (eGFR <45 mL/min).
5. Nephrol Dial Transplant. 2015;30 Suppl 2:ii1-142.
6. 20. National Kidney Foundation. KDOQI Clinical Practice Guideline for Hemodialysis
7. Adequacy: 2015 update. Am J Kidney Dis. 2015;66(5):884-930.
8. 21. Lee VW, Tunnicliffe DJ, Rangan GK. KHA-CARI Autosomal Dominant Polycystic
9. Kidney Disease Guideline: Management of End-Stage Kidney Disease. Semin

426	Nephrol. 2015;35(6):595-602.e12.

427. 22. Grupo de trabajo de la Guía de Práctica Clínica sobre la Detección y el Manejo de la
1. Enfermedad Renal Crónica. Guía de Práctica Clínica sobre la Detección y el Manejo
2. de la Enfermedad Renal Crónica. Ministerio de Sanidad, Servicios Sociales e
3. Igualdad. Instituto Aragonés de Ciencias de la Salud; 2016. Guías de Práctica Clínica
4. en el SNS.
5. 23. Farrington K, Covic A, Aucella F, et al; ERBP guideline development group. Clinical
6. Practice Guideline on management of older patients with chronic kidney disease stage
7. 3b or higher (eGFR <45 mL/min/1.73 m2). Nephrol Dial Transplant. 2016;31(suppl

435	2):ii1-ii66.

436	24. Woodrow G, Fan SL, Reid C, Denning J, Pyrah AN. Renal Association Clinical

437	Practice Guideline on peritoneal dialysis in adults and children. BMC Nephrol.

438	2017;18(1):333.

439	25. NICE guideline. Renal replacement therapy and conservative management. 2018 3rd

440	October.

441	26. Brown EA, Blake PG, Boudville N, et al. International Society for Peritoneal Dialysis

442	practice recommendations: Prescribing high-quality goal-directed peritoneal dialysis.

443	Perit Dial Int. 2020;40(3):244-253.

444. 27. Sekercioglu N, Al-Khalifah R, Ewusie JE, Elias RM, Thabane L, Busse JW, Akhtar-
1. Danesh N, Iorio A, Isayama T, Martínez JP, Florez ID, Guyatt GH. A critical
2. appraisal of chronic kidney disease mineral and bone disorders clinical practice
3. guidelines using the AGREE II instrument. Int Urol Nephrol. 2017;49(2):273-284.
4. 28. Coronado Daza J, Vernooij RWM, Salas K, Osorio D, Urrútia Cuchí G. Quality
5. assessment of clinical practice guidelines for chronic kidney disease: a systematic

450	review. BMC Nephrol. 2019;20(1):192.

451. 29. Vanommeslaeghe F, De Mulder E, Van de Bruaene C, Van de Bruaene L, Lameire N,
1. Van Biesen W. Selecting a strategy for prevention of contrast-induced nephropathy in
2. clinical practice: an evaluation of different clinical practice guidelines using the
3. AGREE tool. Nephrol Dial Transplant. 2015;30(8):1300-6.
4. 30. Lopez-Vargas PA, Tong A, Sureshkumar P, Johnson DW, Craig JC. Prevention,
5. detection and management of early chronic kidney disease: a systematic review of
6. clinical practice guidelines. Nephrology (Carlton). 2013;18(9):592-604.
7. 31. Chan CT, Blankestijn PJ, Dember LM, et al. Conference Participants, author. Dialysis
8. initiation, modality choice, access, and prescription: conclusions from a Kidney
460. Disease: Improving Global Outcomes (KDIGO) controversies conference. Kidney Int.

461	2019;96:37–47.

462	32. Légaré F, Adekpedjou R, Stacey D, et al. Interventions for increasing the use of

463	shared decision making by healthcare professionals. Cochrane Database Syst Rev

464	2018;7:CD006732.

465. 33. Choosing Wisely [Internet]. American Society of Nephrology. Five things physicians
1. and patients should question. 2012 4th April. Available from:
2. <http://www.choosingwisely.org/societies/american-society-of-nephrology/> [Accessed
3. 20^th^ November, 2021]
4. 34. Engels N, de Graav G, van der Nat P, van den Dorpel M, Bos WJ, Stiggelbout AM.
5. Shared decision-making in advanced kidney disease: a scoping review protocol. BMJ

471	Open. 2020;10(2):e034142.

472	35. Salas-Gama K, Díaz JM, Coronado J, et al. Patient decision aids for adults with

473	advanced chronic kidney disease with a medical recommendation to start dialysis: a

474	scoping review protocol [published online ahead of print, 2021 23rd June]. JBI Evid

475	Synth. 2021;10.11124/JBIES-20-00437.

476. 36. Schünemann H, Brożek J, Guyatt G, Oxman A. The GRADE Working Group.
1. Handbook for grading the quality of evidence and the strength of recommendations
2. using the GRADE approach. Updated October 2013.
3. 37. Mustafa RA, Wiercioch W, Arevalo-Rodriguez I, Cheung A, Prediger B, Ivanova L,
4. Ventresca M, Brozek J, Santesso N, Bossuyt P, Garg AX, Lloyd N, Lelgemann M,
5. Bühler D, Schünemann HJ. Decision making about healthcare-related tests and
6. diagnostic test strategies. Paper 4: International guidelines show variability in their
7. approaches. J Clin Epidemiol. 2017;92:38-46.

| 484 | 38. Harwood L, Clark AM. Understanding pre-dialysis modality decision-making: A |
| --- | --- |
| 485 | meta-synthesis of qualitative studies. Int J Nurs Stud. 2013;50(1):109-20. |
| 486 | 39. Alonso-Coello P, Schünemann HJ, Moberg J, Brignardello-Petersen R, Akl EA, |
| 487 | Davoli M, Treweek S, Mustafa RA, Rada G, Rosenbaum S, Morelli A, Guyatt GH, |
| 488 | Oxman AD; GRADE Working Group. GRADE Evidence to Decision (EtD) |
| 489 | frameworks: a systematic and transparent approach to making well informed |
| 490 | healthcare choices. 1: Introduction. BMJ. 2016;353:i2016. |
| 491 | 40. Alonso-Coello P, Oxman AD, Moberg J, Brignardello-Petersen R, Akl EA, Davoli M, |
| 492 | Treweek S, Mustafa RA, Vandvik PO, Meerpohl J, Guyatt GH, Schünemann HJ; GRADE |
| 493 | Working Group. GRADE Evidence to Decision (EtD) frameworks: a systematic and |
| 494 | transparent approach to making well informed healthcare choices. 2: Clinical practice |
| 495 | guidelines. BMJ. 2016;353:i2089. |
| 496 |  |
| 497 | **Supporting information** |
| 498 | S1 Appendix. Search strategy |
| 499 | S2 Table. Excluded CPG with scores in domains 3 or 6 less than 60% in the AGREE-II instrument |
| 500 | S3 Table. Main objective of included high-quality CPGs |
| 501 | S4 Table. Complete text of recommendations related to the start of dialysis |
| 502 | S5 Table. PRISMA 2020 Checklist |

Figure	[Click here to access/download;Figure;Fig 1.docx](https://www.editorialmanager.com/pone/download.aspx?id=30208934&guid=30ee4a13-ab13-4cbc-b436-01f9b819c614&scheme=1)

1

2

3

4

5

6

7

8

9

10

11

12

13

14

15

16

17

18

19

20

21

22

23

24

25

26

27

28

29

30

31

32

34

36

38

39

40

42

44

47^6^

48

49

**Fig 1. PRISMA Flow diagram**

**Identification of CPGs via databases and other methods (Original search – September 2018)**

**Identification of CPGs via databases and other methods (Update search – August 2021)**

Guidelines included in review (n = 11)

Guidelines included in review (n = 1)

*From:* Page MJ, McKenzie JE, Bossuyt PM, Boutron I, Hoffmann TC, Mulrow CD, et al. The PRISMA 2020 statement: an updated guideline for reporting systematic reviews. BMJ 2021;372:n71. doi: 10.1136/bmj.n71. For more information, visit: [http://www.prisma-statement.org](http://www.prisma-statement.org/)

Reports considered for quality assessment with AGREE-II

(n = 4)

Records excluded (n = 7)

Domain 3 and Domain 6 of the AGREE-II instrument <60% (n = 7)

Reports considered for quality assessment with AGREE-II

(n = 18)

Records excluded (n = 3)

Domain 3 and Domain 6 of the AGREE-II instrument <60% (n = 3)

Reports excluded:

(n = 27)

Belong to the same CPG (n=13) CPG does not include recommendations of interest (n = 6) Not English language (n = 4)

Not a CPG (n = 2) Duplicate (n=1)

CPG already included (n=1)

Reports assessed for eligibility (n = 31)

Reports excluded:

(n = 89)

CPG does not include recommendations of interest (n = 36) Not a CPG (n = 18)

Do not describe how the evidence was assessed (n=15)

Reports not retrieved (n = 9) Not English language (n = 5) Belong to the same CPG (n=5) Duplicate (n=1)

Reports assessed for eligibility (n = 107)

Records excluded by title and abstract

(n = 482)

Records screened (n = 513)

Records excluded by title and abstract

(n = 1481)

Records screened (n = 1588)

**Screening**

Records removed *before screening*:

Duplicate records removed (n = 199)

Records identified from: Databases (n = 701) Websites (n = 11)

Records removed *before screening*:

Duplicate records removed (n = 1040)

Records identified from: Databases (n = 2587) Websites (n = 41)

**Identification**

**Included**

Supporting Information

#### [Click here to access/download](https://www.editorialmanager.com/pone/download.aspx?id=30213735&guid=b7eeefd4-4db2-4145-8dad-3ad571fd762d&scheme=1)

[Supporting Information Supporting information.pdf](https://www.editorialmanager.com/pone/download.aspx?id=30213735&guid=b7eeefd4-4db2-4145-8dad-3ad571fd762d&scheme=1)
