# Supplementary material for: Recommendations of high-quality clinical practice guidelines related to the process of starting dialysis: A systematic review
Source: PLoS One. 2022 Jun 13;17(6):e0266202. doi: 10.1371/journal.pone.0266202 (PMC9191707; doi:10.1371/journal.pone.0266202)
Supplement: Attachment — Submitted filename: Response to Reviewers.docx [file pone.0266202.s008.docx]

**RESPONSE TO REVIEWERS**

Dear Prof. dr. Pierre Delanaye

Academic Editor

PLOS ONE

We are grateful to the editor and reviewers for their time and constructive comments on our manuscript. We have carefully reviewed the comments and have revised the manuscript accordingly. We wish to submit a revised version of the manuscript for further consideration in the journal. Changes in the initial version of the manuscript are highlighted in the revised version. Below, we also provide a point-by-point response explaining how we have addressed each subject editor or reviewers’ comments.

We look forward to the outcome of your assessment.

Yours sincerely,

On behalf of the co-authors

Karla Salas-Gama

Corresponding author

PONE-D-21-37316

Recommendations of high-quality clinical practice guidelines related to the process of starting dialysis: a systematic review.

**Editor comments**

We have ensured that our manuscript meets PLOS ONE’s requirements (style requirements, file naming, competing interest statement, title page and reference list).

**Reviewers’ comments**

**Reviewer 2**

- In the end, only CPGs in English and Spanish were included. It might be clearer to simply make that statement upfront so that the reader does not have to wait until Table 1 to know if a CPG in any another language has been included.

Our answer: Thank you for this suggestion. We have modified the statement to be more explicit (lines 75-76)

- ‘Continuous automatic PD” is not standard terminology. Consider “automated PD” or “cyclic PD”.

Our answer: We have changed the terminology to “automated PD.” (line 88)

- Typo corrections and written suggestions

Our answer: Accepted (lines 102, 216, 218, 226, 227, 245, 247, 248, 258, 265, 266, 267, 269-271, 276, 279, 280, 341, 346, 347, 348, 349, 363)

- Table 4 shows that those CPGs that did not make their statement a strong recommendation actually did not make it a weak one either. They simply did not grade it. Consider making this clear in the text as well.

Our answer: Thank you for this comment. We have specified the number of CPGs that graded and not graded the statement (lines 234-235)

- What matters here is what the recommendations are about, not to whom they are directed (which is implicit). Please consider either removing “directed to healthcare professionals” or putting this segment between two commas for more clarity.

Our answer: Thank you for this suggestion. We have removed that fragment of a sentence (line 280)

- According to the Methods section, the assessment of selected CPGs was done by 2 reviewers (lines 127-132 of this document). A third one was involved only upstream at the selection step in case of a disagreement about whether a given CPG should be selected or not (lines 114-117). ICC values were usually excellent (> 0.90) but could you please briefly discuss how much you might have improved the reliability of your assessment by having 3 or (as recommended in the AGREE II User Guide) 4 reviewers, especially for those CPGs for which the ICC was below 0.90?

Our answer: Thank you for this suggestion. We have added this point as a limitation (lines 325-327).

- Two other potential limitations might warrant a short discussion. First, Table 3 does not provide overall assessment scores. Second, no mention is made either in the table or in the text about your recommendations for use of individual CPGs. Both steps are expected as per the AGREE II User Guide

Our answer: Thank you for this comment. We think this is not a limitation because the main purpose of using the AGREE II instrument was to assess the methodological quality of CPGs across the six domains. Nevertheless, we have this information, and we can provide it as a Supporting information of the manuscript. Please see S3 Table. Complete scores of high-quality CPGs according to the AGREE II instrument of the Supporting information document.

**Reviewer 3**

- In the introduction, I suggest to not overinterprated the paper of Korevaar and coll (ref4) since this RCT was not completed.

“A randomised controlled trial [4] compared starting dialysis with HD vs PD showed no differences in mortality at two years. The results suggested a hypothesis of equivalence between PD and HD.”

Our answer: Thank you for this comment. We have modified our statement to avoid overinterpreting results (lines 49-52).
